# Synergistic Action of Corn, Wolf Fruit, and Butterfly Lily Starches in Bioactive Coatings and Their Potential Application in the Physiological Quality of Common Beans

**DOI:** 10.3390/polym18111378

**Published:** 2026-06-02

**Authors:** Ana Maria Gomes Batista, Diego Palmiro Ramirez Ascheri, Itamar Rosa Teixeira, Roberta Signini, Rejane Dias Pereira Mota, José Luis Ramírez Ascheri

**Affiliations:** 1 Stricto Sensu Graduate Program in Agricultural Engineering (CPPGEA) of the State University of Goiás (UEG), Central Campus—Headquarters: Anápolis—CET Henrique Santillo, BR-153, nº 3.105, Fazenda Barreiro do Meio, Anápolis CEP 75132-903, GO, Brazil; anaana.amg@gmail.com (A.M.G.B.); itamar.texeira@ueg.br (I.R.T.); 2 Stricto Sensu Graduate Program in Molecular Sciences (PPGCM) of UEG, Anápolis CEP 75132-903, GO, Brazil; roberta.signini@ueg.br; 3 Federal Institute of Goiás, Anápolis Campus, Anápolis CEP 75131-457, GO, Brazil; rejane.mota@ifg.edu.br; 4 Food Extrusion Pilot Plant and Cereal Quality Laboratory, Embrapa Agroindústria de Alimentos, Av. das Américas 29501, Guaratiba, Rio de Janeiro CEP 23020-470, Brazil; jose.ascheri@embrapa.br

**Keywords:** *Zea mays*, *Solanum lycocarpum*, *Hedychium coronarium*, *Phaseolus vulgaris*, bio-based polymers, starch films, seed coating, physiological seed quality, sustainability

## Abstract

This study aimed to characterize corn (CS), wolf fruit (WF), and butterfly lily (BL) starches; to develop bioactive coatings from pure starches and their binary and ternary blends; and to evaluate the synergistic effects of these formulations on the physiological quality of common bean seeds. Films were prepared by thermocompression (80 °C, 6 min, 3 t) of film-forming solutions obtained via microwave processing and formulated using a simplex-centroid mixture design. The starches were characterized in terms of amylose content, Scanning Electron Microscopy, Fourier Transform Infrared Spectroscopy, Differential Scanning Calorimetry, Rapid Visco Analyser, while the films were evaluated for thickness, water solubility, and water vapor permeability. The film-forming solutions were applied as coatings, and seed physiological quality was assessed through germination, first count, seedling length, and dry mass. BL exhibited higher gelatinization temperatures and produced films with adequate thickness and moderate permeability, indicating greater structural stability. The CS:BL blend produced films with balanced hydration, promoting rapid and uniform water uptake. Coatings based on BL and CS:BL showed the highest germination percentages, whereas CS:WF resulted in lower physiological performance. These results demonstrate that film properties directly influence seed vigor and germination. BL, alone or blended with CS, represents a promising starch-based material for seed coating, promoting high physiological quality and environmentally friendly characteristics.

## 1. Introduction

The savanna is the second-largest Brazilian biome, harboring endemic species that can be exploited as natural sources to produce starch-based materials, such as starch—an emerging polymer with great potential to produce starch-based films and film-forming solutions. These materials are widely used in coatings for fruits and seeds and can replace conventional starches and other synthetic materials due to their low cost and wide availability. However, although corn starch (CS) is commonly available on the market, its application as a coating agent may face competition and increased costs due to high demand from other industries, such as pharmaceuticals, food, biofuels, and chemicals.

Wolf fruit (*Solanum lycocarpum*) and butterfly lily (*Hedychium coronarium*) are species found in the Brazilian savanna, with the potential to produce low-cost starches with functional properties like those of conventional starches. When utilized, these starches can reduce the demand for traditional sources in the development of starch-based materials. The wolf fruits can reach diameters ranging from 7 to 16 cm, with an average weight of 750 g [1], and exhibit high starch content and functional properties comparable to those of corn starch. The rhizomes of butterfly lily contain starch that can be used in the production of starch-based materials, with granules showing high resistance to gelatinization, an important parameter in film development.

These native starches are believed to be capable of partially replacing corn starch. Their blends can improve film-forming properties and yield high-quality films, as well as coatings capable of covering the surface of bean seeds, forming a continuous layer and contributing to improved physiological quality of the treated seeds. These properties are related to the behavior of starch, which, when in aqueous suspension and subjected to high temperatures, tends to gelatinize, forming a highly viscous and adhesive paste [2].

Blends of starches from different botanical sources can significantly affect the phy-sicochemical and functional properties of their derivatives [3,4]. In coatings, starches with high viscosity tend to produce thicker and more cohesive layers [5], which can enhance effectiveness by protecting the seeds and providing a favorable environment for their development. In contrast, starches with low viscosity may result in thinner and less uniform coatings, potentially compromising their effectiveness.

The selection of a native starch with the potential to replace conventional starch depends primarily on its viscosity, a key factor influencing its performance as a seed coating agent. This characteristic may result in greater protective capacity, as well as improved physiological and sanitary quality, ensuring more vigorous seedlings and uniform stands [6]. Productivity can be increased when this technique performs well under adverse biotic and abiotic stress conditions [7]. To this end, it is essential to evaluate physiological potential after seed treatment by assessing seed performance after sowing and ensuring proper seedling development in the field [8].

This study aimed to characterize corn, wolf fruit, and butterfly lily starches; to develop bioactive coatings from pure starches and their binary and ternary blends; and to evaluate the synergistic effects of these formulations on the physiological quality of common bean seeds.

## 2. Materials and Methods

### 2.1. Material

Wolf fruits and butterfly lily rhizomes were collected from healthy plants located along the banks of the Estrema River at the State University of Goiás (Central Campus—Headquarters: Anápolis—CET) (latitude 16°23′02″ S, longitude 48°56′37″ W, altitude 1141 m), in Goiás, Brazil. Green fruits and rhizomes of different sizes were used for starch extraction. Commercial CS was purchased from an agricultural supply store in Anápolis, GO, Brazil, with 15% moisture content (dry basis) and 85% purity. Common bean seeds of the cultivar BRS Estilo were provided by the Plant Analysis Laboratory of the State University of Goiás (Anápolis, GO, Brazil).

### 2.2. Starch Extraction and Purification Process

Individually, the fruits and rhizomes were washed, peeled, chopped, and ground in an industrial blender (Metvisa Metalúrgica^®^, LQ.25, Brusque, SC, Brazil) with abundant potable water containing 5 g L^−1^ sodium metabisulfite (Synth, Diadema, SP, Brazil) to prevent browning [9]. The starches were separated from the fibers by filtration through polyester fabric, followed by successive sieving through stainless steel sieves (Bertel, São Paulo, SP, Brazil) with mesh openings ranging from 75 to 250 μm. The material was subsequently decanted, washed with absolute ethanol (Synth, Diadema, SP, Brazil) to remove lipids and excess water, and dried in a forced-air oven (Solab^®^, model SL-102, Piracicaba, SP, Brazil) at 45 °C for 14 h.

### 2.3. Film Processing

The proportions of pure starches and their blends were defined using a simplex-centroid mixture design [10] to evaluate the effect of blend composition on the response variables [thickness (δ), water solubility (WS), and water vapor permeability (WVP)]. The proportions of each starch (XCS, XWF, and XBL) were treated as coded variables ranging from 0 to 1, with the constraint XCS + XWF + XBL = 1. The design consisted of seven experimental points: three pure components (1,0,0; 0,1,0; 0,0,1); three binary blends (1/2,1/2,0; 1/2,0,1/2; 0,1/2,1/2); and one ternary centroid (1/3,1/3,1/3). All experiments were performed in triplicate, totaling 21 runs, conducted in random order.

The films were prepared from aqueous solutions containing 57.14% (*w*/*v*) of pure starches or their blends, to which 1.6 g of glycerol was added. The solutions were mixed in 10 mL glass vials (48 mm × 22 mm) and pre-gelatinized for 20 s in a conventional microwave oven [11] (Panasonic, model NN-ST65HWRU, Beijing, China) at maximum power (2450 MHz, 900 W), producing film-forming pastes.

The films were produced by thermal compression of the pastes [11], applying a pressure of 3 t using a hydraulic press (Tecnal, TE-098-E6, Piracicaba, SP, Brazil), equipped with thermocouples to control the heating plates at 80 °C for 6 min. After pressing, the films were stored in parchment paper envelopes inside a desiccator containing a magnesium nitrate solution (51% relative humidity at 26 ± 1 °C) for subsequent characterization.

### 2.4. Physical and Physicochemical Characterization of Starch

#### 2.4.1. Starch Granule Morphology and Size Distribution

Micrographs of the starches were obtained using a TM3000 scanning electron microscope (SEM) (Hitachi, Tokyo, Japan) at an accelerating voltage of 15 kV and a magnification of 1.0k× [11]. For each sample, approximately 50 starch granules were observed and measured using ImageJ software (IJ 1.46r, Bethesda, MD, USA). For irregularly shaped granules, the largest dimension was considered as the diameter.

#### 2.4.2. Apparent Amylose Content of Starch (AAC)

The apparent amylose content was determined using the iodine colorimetric method described in [12]. Starch samples (0.1 g) were dispersed in 2 mL of 0.1 mol L^−1^ NaOH and heated at 80 °C for 30 min. After cooling, the pH was adjusted to 3.5 using 0.1 mol L^−1^ HCl and citrate-phosphate buffer (pH 3.2), and the volume was made up to 50 mL with distilled water. An aliquot of 2 mL of iodine solution (0.2% I_2_/2% KI) was added, and the mixture was allowed to stand for 15 min in the dark. Absorbance was measured using an SP-2000UV spectrometer (Spectrum, Shanghai, China) at 620 nm. A calibration curve was prepared using pure amylose and amylopectin standards (0–100% amylose).

#### 2.4.3. Fourier Transform Infrared Spectroscopy (FTIR) Analysis

FTIR spectra were obtained using a PerkinElmer infrared spectrophotometer (Spectrum Frontier FT-IR/NIR; PerkinElmer, Norwalk, CT, USA) in the spectral range of 4k–0.4k cm^−1^, with a resolution of 4 cm^−1^. The samples were previously dried in an oven at 60 ± 1 °C for 12 h, ground, and mixed with KBr at a ratio of 1:100 (m/m) [13]. Peak intensities corresponding to the main functional groups of starch were determined, and the crystallinity index was calculated based on the bands at 1047 and 1022 cm^−1^, defined as R_1047/1022_ = absorbance intensity at 1047 cm^−1^ divided by absorbance intensity at 1022 cm^−1^ [14].

#### 2.4.4. Differential Scanning Calorimetry (DSC) Analysis

Calorimetric analysis was performed using a differential scanning calorimeter (DSC), model Q200 (TA Instruments, New Castle, DE, USA). The instrument was calibrated u-sing indium as a standard. For the determination of starch gelatinization enthalpy, approximately 5 mg of sample with known moisture content was placed in an aluminum pan, which was hermetically sealed. The scanning profile consisted of equilibration at 5 °C, followed by heating to 110 °C at a heating rate of 10 °C min^−1^ under a nitrogen flow of 50 mL min^−1^. The gelatinization enthalpy was calculated using Universal Analysis software, version 4.3a [2]. The following parameters were determined from the DSC thermograms: onset temperature (To), peak temperature for gelatinization (Tp), conclusion temperature (Tf), enthalpy (ΔH), and melting temperature for amylose–lipid complex dissociation ($T_{m}$).

#### 2.4.5. Rapid Visco Analyser (RVA) Measurement Conditions 

A rapid visco analyzer (RVA 4, Newport Scientific Pty Ltd., Warriewood, Australia) was used to measure the apparent viscosity of the samples, following the standard method described in the instrument manual. A starch sample (10% *w*/*w*, dry basis) was subjected to the following heating and cooling cycle: initially held at 25 °C for 2 min; heated at approximately 13 °C min^−1^ to 95 °C; held at 95 °C for 3 min; and then cooled at approximately 13 °C min^−1^ to 25 °C, where it was maintained until the end of the test (20 min). The viscosity parameters evaluated were pasting temperature (PT), peak viscosity (PV, maximum viscosity during heating), breakdown (BD, difference between peak viscosity and hot paste viscosity), and setback (SB, difference between viscosity during cooling and hot paste viscosity) [2].

### 2.5. Physical Characterization of Films

Film thickness was measured using a digital external micrometer (ISO-MASTER^®^, Tesa, Switzerland) with a precision of 0.01 mm. Water solubility (WS, %, *w*/*w*) was determined according to the method described by [15]. Water vapor permeability (WVP, g m^−1^ s^−1^ Pa^−1^) was determined using the desiccant method, following ASTM standard E96-95 [16].

### 2.6. Common Bean Seed Coating Process

In the common bean seed coating process [17], starch solutions and their blends (4 g) were used as coating agents, together with glycerol (1.6 g) and distilled water up to a final volume of 100 mL. The solutions were gelatinized at 85 °C for 5 min. Subsequently, three drops of 0.5% potassium sorbate solution were added as an antimicrobial agent. The experimental design was the same as that used for the preparation of the starch-based films, with four replications.

The experiment was conducted in 150 mL beakers containing 200 common bean seeds, to which the coating solution was added, and the seeds were left to stand for 5 min. The treated seeds were then poured onto a stainless-steel mesh to remove excess liquid and dried in a forced-air oven at 35 °C for 1 h. After coating, the seeds were immediately subjected to physiological quality evaluation.

### 2.7. Physiological Quality Tests of Seeds

#### 2.7.1. Seed Germination Test (GER)

The GER was conducted to evaluate seed viability. For each treatment, 200 seeds were divided into four replicates of 50 seeds per experimental unit and placed on Germitest paper previously moistened with distilled water at a volume equivalent to 2.5 times the dry paper weight. The paper rolls were then placed in a germination chamber at 25 °C. Evaluation was performed on the 8th day of the experiment, considering only the percentage of normal seedlings [18].

#### 2.7.2. First Count (FC)

The FC was performed in conjunction with the GER to assess seed vigor, based on the percentage of normal seedlings evaluated on the 5th day of the germination test [18].

#### 2.7.3. Seedling Length (SL)

Seedling length was evaluated by sowing four subsamples of 10 seeds per treatment on Germitest paper moistened with water at 2.5 times the dry paper weight. The rolls were incubated in a germination chamber at 25 °C for 10 days. Seedling length was measured with a ruler and expressed in centimeters [19].

#### 2.7.4. Seedling Dry Mass (SDM)

For the SDM, only normal seedlings obtained from the seedling length test were eva-luated [19]. The replicates from each repetition were placed in labeled paper bags and dried in a forced-air oven at 80 °C for 24 h. After this period, each replicate was weighed using a balance with a precision of 0.001 g, and the mean results were expressed as milligrams per seedling.

### 2.8. Statistical Analysis

Granule size distribution data were subjected to frequency analysis at a significance level of *p* < 0.05, and normality was assessed using the Shapiro–Wilk test. Results were expressed as mean ± standard deviation (SD).

Second-order polynomial models involving blends were fitted to the response variables δ, WS, and WVP, as well as to the results of the common bean seed quality tests. The significance of the models and their coefficients was evaluated by analysis of variance (ANOVA) (*p* < 0.05). Model adequacy was assessed using the adjusted coefficient of determination ($R_{adj.}^{2}$) and lack-of-fit tests. Contour plots were generated to visualize the effect of composition on the responses. All analyses were performed using Statistica 14.1.0.8 software (Tulsa, OK, USA) [20], and graphs were generated using OriginPro 2026 (Northampton, MA, USA).

## 3. Results and Discussion

### 3.1. Physical, Physicochemical, Structural, and Pasting Properties of Starch

#### 3.1.1. Morphology and Distribution of Granule Diameter

**Figure 1:**
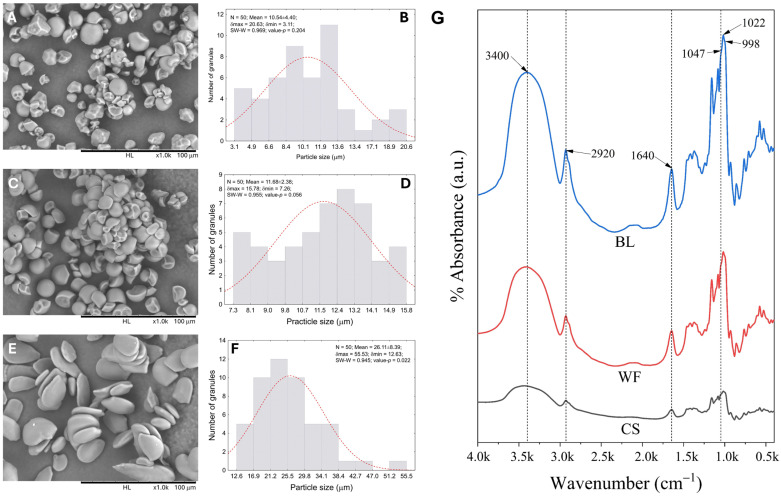
Microscopic properties (SEM, 1000× magnification) and spectroscopic analysis and granule size distribution of: (**A**,**B**) corn starch (CS); (**C**,**D**) wolf fruit starch (WF); (**E**,**F**) butterfly lily starch (BL); and (**G**) FTIR spectra of CS, WF, and BL.

Micrograph analyses of CS, WF, and BL starches (Figure 1A, Figure 1C, and Figure 1E, respectively) revealed granules with typical morphologies. CS granules exhibited polygonal and rounded shapes with smooth surfaces and no visible fissures, characteristics of native starch. WF, in turn, showed granules with truncated ellipsoidal and irregular shapes, presenting a concave base. BL granules were predominantly asymmetrical ellipsoids with curved, irregular triangular, and flattened shapes [11].

The Shapiro–Wilk test for granule size distribution of CS and WF (Figure 1B and Figure 1D, respectively) indicated normal distribution (W = 0.969; *p* = 0.204 and W = 0.955; *p* = 0.120, respectively), whereas BL (Figure 1F) showed an asymmetric distribution (W = 0.945; *p* = 0.022), likely due to its broader size range (12.63–55.53 µm) and the presence of two distinct granule populations. In contrast, the narrower ranges of CS and WF (3.11–20.63 µm and 7.26–15.78 µm, respectively) favored adherence to a normal distribution.

The mean granule diameters of CS and WF were 10.54 ± 4.40 µm and 11.68 ± 2.38 µm, respectively, whereas BL showed a mean value of 26.11 ± 8.39 µm (Table 1), all of which were lower than those reported in the literature [11].

#### 3.1.2. Apparent Amylose Content

BL exhibited the highest AAC (Table 1), with a value of 33.9 ± 1.2%, higher than those determined for CS (25.5 ± 0.6%) and WF (22.7 ± 0.4%). The AAC values obtained for both CS and WF fall within the typical range reported for cereal starches (21–28%) [11,21,22]. Despite both starches lying within this range, their functional behavior may differ due to other structural factors beyond AAC, such as granule morphology, amylopectin fine structure, and molecular weight distribution. Nevertheless, the intermediate AAC of CS suggests moderate gelatinization and retrogradation properties, positioning it as a material with balanced functionality for food applications.

The relatively low AAC of WF (22.7 ± 0.4%) and, consequently, its high amylopectin content confer potential for applications requiring high viscosity and low tendency to retrogradation, such as sauces and frozen instant soups, where stability during refrigerated storage is crucial. In addition, amylopectin-rich starches are valued for their ability to form clear and stable pastes [22,23].

The notable differences in AAC values and granule size confer BL a decisive distinction in its functional behavior as a film-forming and seed coating agent. High-amylose starches tend to produce pastes with greater retrogradation [24], while the presence of very small (<5 µm) or very large (>18 µm) granules directly influences properties such as gelatinization temperature and paste viscosity.

#### 3.1.3. FTIR Spectra and Functional Group Analysis 

Mid-infrared spectroscopy (Figure 1G) was used to evaluate the structure of CS, WF, and BL starches, allowing the identification of conformational changes at the molecular level, as well as providing information on short-range order and hydrogen-bond interactions within the granules [25,26,27,28].

The bands at 3400 cm^−1^, 2920 cm^−1^, and 1640 cm^−1^ are attributed, respectively, to O–H stretching vibrations, reflecting extensive intra- and intermolecular hydrogen-bonding networks characteristic of the granular starch structure; to C–H stretching vibrations associated with hydrogen atoms in the glucose ring methyl groups; and to the bending vibration of bound water [28].

The region between 1200 and 900 cm^−1^ is dominated by skeletal vibrations involving C–O, C–O–C, and C–C stretching, as well as α-(1→4) glycosidic linkages between 1150 and 1023 cm^−1^ [27,28,29]. In the present study, all samples exhibited significant absorbance in this region, confirming the integrity of the glucan chains.

The band at 998 cm^−1^ is associated with skeletal vibrations of the glucose ring and is sensitive to starch chain conformation. This band is attributed to C–O–H vibrations and is correlated with the amorphous state of starch [25,26,28]. In this region, differences in intensity were observed among the studied starches, with a more pronounced shoulder in WF.

The band at ~1047 cm^−1^ is commonly associated with ordered regions of starch, related to double-helix formation and crystalline domains, whereas the band at ~1022 cm^−1^ corresponds to amorphous regions, reflecting disordered chains and higher molecular mobility [14]. These bands were used to calculate the R_1047/1022_ ratio, an empirical indicator of short-range molecular order in starches, reflecting the balance between ordered and disordered domains [14,25,26,28].

The R_1047/1022_ values obtained for the three samples ranged from 0.928 to 0.946 (Table 1), indicating only minor differences in short-range molecular organization among the starches. CS showed the highest ratio (0.946), suggesting a more ordered short-range structure compared to the other samples. This result is consistent with cereal starches, which typically exhibit A-type crystallinity [11,28,30], characterized by dense packing of amylopectin double helices [28]. The higher relative absorbance at 1047 cm^−1^ indicates a greater proportion of ordered domains compared to amorphous regions in the native granule structure.

WF exhibited the lowest ratio (0.929), indicating reduced short-range order. This is particularly interesting, as low amylose content (22.7%) would not necessarily be expected to correspond to reduced structural order. This behavior can be explained by the fine structure of its amylopectin: the predominance of chains with a degree of polymerization between 13 and 24 (B1 chains) [31] may lead to the formation of smaller or less stable crystalline clusters, resulting in a more amorphous granule at the nanometric scale. The FTIR results, particularly the more pronounced band at 1022 cm^−1^, support this interpretation, indicating lower short-range structural organization in WF granules.

BL exhibited an intermediate ratio (0.931), very close to that of WF. Considering its high apparent amylose content (33.9%), a more pronounced structural disorder and thus a lower ratio would be expected. However, its granular morphology (asymmetrical ellipsoids with eccentric hilum and radial lines, similar to potato starch) suggests the presence of very long chains (DP > 37) in amylopectin, typical of B-type starches [11], such as ginger starch [32]. These long chains act as structural elements spanning multiple clusters, contributing to stability and maintaining a reasonable degree of short-range order, even at high amylose levels.

These results are consistent with the literature [28], which demonstrates that starch short-range order does not depend solely on amylose content, but rather on the complex interaction between amylose content and amylopectin molecular architecture. Furthermore, they support the idea that the R_1047/1022_ ratio should be interpreted as a tool for identifying relative ordering trends rather than as an absolute measure of crystallinity [14].

#### 3.1.4. Thermal Properties of Starch

**Figure 2:**
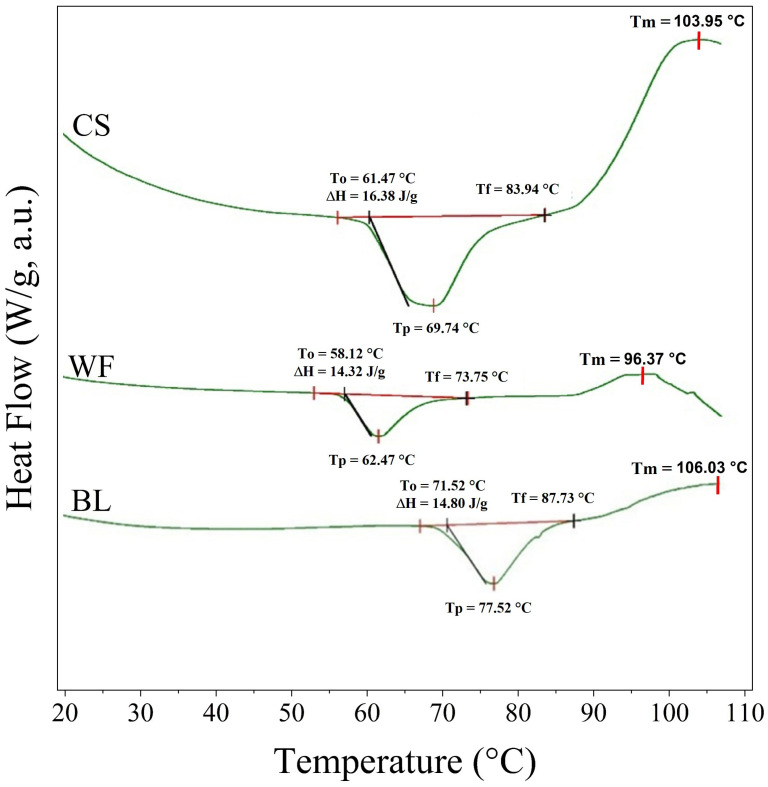
DSC thermograms of corn starch (CS), wolf fruit starch (WF), and butterfly lily starch. The red horizontal lines represent the linear baselines drawn between To and Tf for calculation of enthalpy (ΔH). Tm is the Menting peak.

The thermal properties of the starches were analyzed by differential scanning calorimetry (DSC), which records endothermic events associated with starch gelatinization. The numerical parameters are presented in Table 1, and the corresponding DSC thermograms are shown in Figure 2.

BL exhibited the highest gelatinization temperatures (To = 71.52 °C; Tp = 77.52 °C; Tf = 87.73 °C), indicating greater resistance to hydration and heat-induced disruption of the granular structure. These results are consistent with the thermal behavior reported for butterfly lily starch in the literature [2] (To = 73.10 °C; Tp = 79.22 °C; Tf = 99.9 °C) and for ginger starch [33,34] (To = 65.8 °C; Tp = 75.4 °C; Tf = 85.2 °C). This behavior can be attributed to its large granule size (12.6–55.5 µm), since larger granules offer greater resistance to water penetration and heat diffusion, delaying gelatinization. In addition, the high amylose content (33.9%) may contribute to the formation of a more compact and organized internal structure, requiring higher thermal energy to initiate swelling and molecular disruption.

In contrast, WF exhibited the lowest gelatinization temperatures (To = 58.12 °C; Tp = 62.47 °C; Tf = 73.75 °C), reflecting greater thermal susceptibility. These values differ from those reported by other authors [35] for wolf fruit starch, with endothermic transition temperatures of To = 61.25 °C, Tp = 64.5 °C, and Tf = 67.5 °C. These discrepancies may be related to factors such as fruit maturation stage at harvest, edaphoclimatic conditions, or differences in extraction and analytical protocols. The thermal behavior observed in this study is directly associated with the smaller granule size of WF (7.3–15.8 µm) and its more homogeneous distribution. Smaller granules have a higher specific surface area, which facilitates water uptake and heat transfer, resulting in earlier gelatinization. The low amylose content (22.7%) and the consequent predominance of amylopectin also contribute to this behavior, since amylopectin crystalline regions, although present, are less thermally stable.

CS exhibited intermediate gelatinization temperatures (To = 61.47 °C; Tp = 69.74 °C; Tf = 83.94 °C), values consistent with those reported in the literature [36,37,38] and coherent with its moderate amylose content (25.5%) and broad granule size distribution (3.1–20.6 µm). The wide variability in granule size explains the broad gelatinization range (Tf − To = 22.47 °C), the largest among the three samples analyzed. Smaller granules gelatinize at lower temperatures, whereas larger ones require higher temperatures for complete structural disruption, resulting in a broadened endothermic peak in the thermogram.

The enthalpy of gelatinization (ΔH) reflects the energy required to melt amylopectin crystallites and disrupt the granular structure, being directly associated with the amount and perfection of crystalline regions. CS showed intermediate and consistent ΔH values (15.65–16.38 J/g), in agreement with its higher FTIR crystallinity ratio (0.946) among the samples, indicating a predominance of ordered and crystalline structures. The narrow ΔH range suggests uniformity in crystal quality within the granules.

WF exhibited the lowest ΔH (14.32–14.76 J/g), indicating that its amylopectin crystallites are less stable or present in lower amounts. This result is consistent with its FTIR ratio (0.928) and low amylose content, which, although favoring amylopectin prevalence, does not result in crystals as well-ordered as those in corn starch. The low enthalpy confirms the lower energy required to disrupt its granules, in agreement with its lower gelatinization temperatures.

BL showed the highest ΔH variation (14.80–24.20 J/g) and the highest maximum value among the samples, consistent with literature reports [11] of 16.16 J/g. This behavior is particularly interesting, as it contrasts with its lower FTIR ratio (0.937), which suggests a predominance of amorphous regions. This apparent contradiction can be explained by granule heterogeneity (12.6–55.5 µm), which leads to different thermal behaviors within the same sample, broadening the ΔH range, as well as by its high amylose content (33.9%), which may form organized structures such as double helices [39,40]. These structures, although not necessarily detected as crystalline order by FTIR ratios, require significant energy to be disrupted during gelatinization.

The DSC thermograms revealed an additional exothermic peak between 90 and 110 °C (Table 1 and Figure 2), attributed to the interaction between leached amylose and endogenous lipids present in native starch granules (Type I) due to the less ordered structure, related to the randomly distributed single intrahelical starch–lipid complex and the short-chain lamellae-like semicrystalline structure [41].

WF exhibited the lowest Tm (96.37 °C), followed by CS (103.95 °C) and BL (106.03 °C). This variation indicates differences in the thermal stability of the amylose-lipid complexes among the botanical sources. The lower Tm observed for WF suggests the presence of shorter-chain or unsaturated lipids, which form less stable complexes and dissociate at lower temperatures. In contrast, the higher Tm values recorded for CS and BL, especially BL (106.03 °C), indicate longer-chain or saturated lipids, which confer greater thermal stability to the complexes, requiring higher temperatures for their dissociation.

#### 3.1.5. RVA Pasting Profiles of the Starches

**Figure 3:**
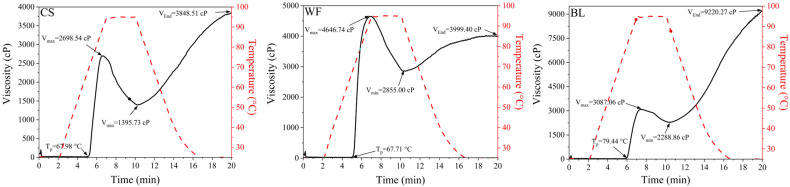
Pasting profiles of corn starch (CS), wolf fruit starch (WF), butterfly lily starch (BL). Solid lines represent viscosity (left Y-axis, cP). The red dashed line represents the temperature profile (right Y-axis, °C).

PT values obtained from the RVA (Figure 3) ranged from 67.98 °C to 79.44 °C, with BL standing out as it exhibited the highest PT among the starches, indicating greater resistance of its granules to hydration and initial swelling (Table 1). This behavior is consistent with its high To value (71.52 °C) observed in DSC and can be explained by its larger granule size (12.6–55.5 µm) and high AAC (33.9%), both of which confer greater structural rigidity and thermal stability to the granules [11,42]. Previous studies have shown that larger granules with higher AAC are more resistant to swelling and require more energy for gelatinization, which is reflected in both RVA and DSC thermal parameters [28,33].

PV represents the maximum swelling capacity of starch granules before rupture. WF exhibited the highest PV (4646.74 cP), a value markedly higher than those observed for CS (2698.54 cP) and BL (3087.06 cP). This behavior is typical of starches with low AAC (22.7%) and, consequently, high amylopectin content, whose highly branched structure favors rapid and extensive granule swelling, resulting in high PV [11,27,28,33]. The high PV of WF is also associated with its smaller granule size (7.3–15.8 µm), which provides a larger surface area for water absorption [11,27,28].

WF also exhibited the highest BD (1791.74 cP), indicating that although its granules swell extensively, they form weak gels that readily collapse, resulting in pastes with low thermal and mechanical stability [43]. This behavior is consistent with its low gelatinization enthalpy (ΔH = 14.32–14.76 J/g) and low gelatinization temperatures observed by DSC, confirming the reduced structural resistance of its granules. In contrast, BL showed the lowest BD (798.2 cP), demonstrating high paste stability under shear stress, which is associated with its higher amylose content and higher gelatinization temperatures, conferring greater rigidity to swollen granules. These results corroborate literature findings [44,45], which relate breakdown in starches to paste fragility and structural stability under shear.

BL exhibited a high SB (6931.41 cP), approximately 2.8 times higher than that of CS (2488.78 cP) and six times higher than that of WF (1144.40 cP). This behavior is directly related to its high AAC (33.9%), which promotes rapid and extensive reassociation of linear chains during cooling, forming rigid gels with a high tendency toward syneresis. It is noteworthy that the setback values obtained in the present study are higher than those reported in the literature, particularly for BL, whose SB was approximately 3.3 times greater [11].

### 3.2. Physical Properties of Starch Films

**Figure 4:**
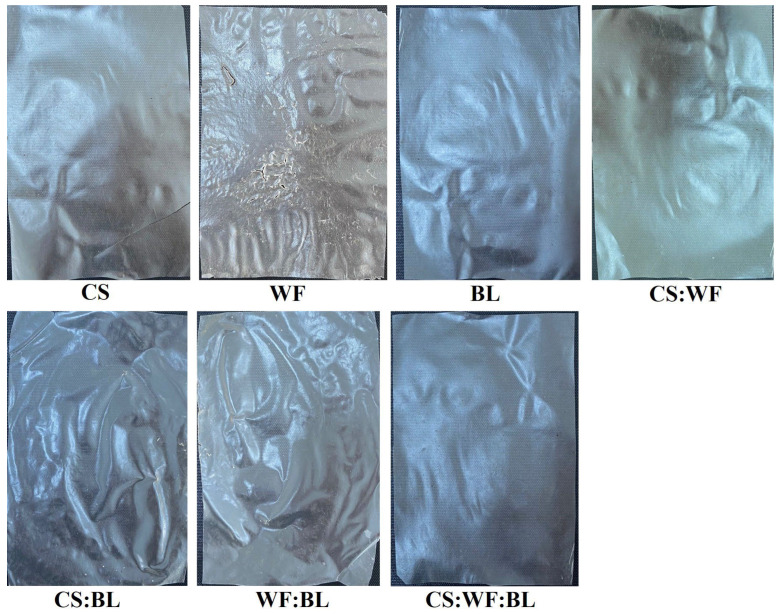
Photographs of films obtained by thermopressing filmogenic starch/glycerol from corn starch (CS), wolf fruit starch (WF), butterfly lily starch (BL), and their blends CS:WF, CS:BL, WF:BL, and CS:WF:BL.

Images of the films are shown in Figure 4, and their δ, WS, and WVP results in Table 2. The ANOVA results are presented in Table 3.

In general, the thermopressed films are relatively thin and malleable, easily conforming to the surface that contains them, as can be clearly observed in the CS:WF film, which shows the imprint of a palm after handling. Except for WF film, the other films exhibited a relatively smooth surface, ranging from transparent to slightly opaque, with a low tendency to form visible crystals. The WF film was very thin, adhering to the Teflon and hindering its removal, resulting in a rough, rugged, and opaque film, probably due to the size of its granules, which is similar to that of CS. Visually, no physical differences were identified between the binary blend CS:WF and the ternary blend CS:WF:BL films when compared to the CS and BL films. The CS:BL and WF:BL blend films are noticeably thin and rough, yet relatively transparent. All blend films exhibited a uniform surface with no phase separation, suggesting good interaction among the starches, likely mediated by glycerol during thermopressing.

The models fitted for δ and WS showed excellent predictive capability, with a non-significant lack of fit (F ≤ 1.86; *p* > 0.05), confirming that the obtained regression equations adequately represent the variation of the experimental data within the studied domain. In contrast, the model fitted for WVP exhibited a significant lack of fit (F = 17.47; *p* = 0.003), indicating that the quadratic model may not be fully adequate to describe this property. This suggests the need for further investigations, including the incorporation of higher-order (cubic) terms in the blend model or the inclusion of additional variables capable of capturing the complexity of the interactions involved in the formation of the polymer matrix and their influence on permeability.

Notably, although the total polymer concentration (57.17% of blend) and glycerol content (1.6 g) were kept constant in the present experimental design, both variables are known to significantly affect water vapor permeability. The polymer-to-water ratio influences the viscosity, drying kinetics, and degree of polymer chain entanglement during film casting, directly affecting matrix density and free volume [46]. Glycerol, as a hydrophilic plasticizer, enhances segmental mobility and typically increases WVP by facilitating water diffusion [47]. Furthermore, the interaction between glycerol and the blend composition may be non-additive, meaning that the optimal plasticizer concentration likely depends on the proportions of components CS, WF, and BL. Therefore, future studies should systematically vary the total solids concentration (e.g., 40–70 g/100 mL) and glycerol content (e.g., 0.5–2.5 g) alongside the blend proportions, employing response surface methodology with cubic or interaction terms to adequately model WVP and fully capture the physicochemical mechanisms governing permeability in this ternary polymer matrix.

#### 3.2.1. Film Thickness

The δ ranged from 39.7 ± 0.6 μm to 64.3 ± 1.2 μm (Table 2) among the different treatments. The WF film exhibited the lowest δ (39.7 μm), which may be related to its lower *δmax* (7.3–15.8 μm) and its greater packing ability during film formation, resulting in a more compact and thinner matrix. In contrast, BL-based films exhibited higher δ values, especially when combined with CS (CS:BL, δ = 50.3 μm) or WF (WF:BL, δ = 49.5 μm), as well as BL alone (δ = 52.7 μm). This behavior is attributed to the higher *δmax* of BL (12.6–55.5 μm) and its high AAC (33.9%), which favor the formation of thicker and more structured matrices during gelatinization and drying.

**Figure 5:**
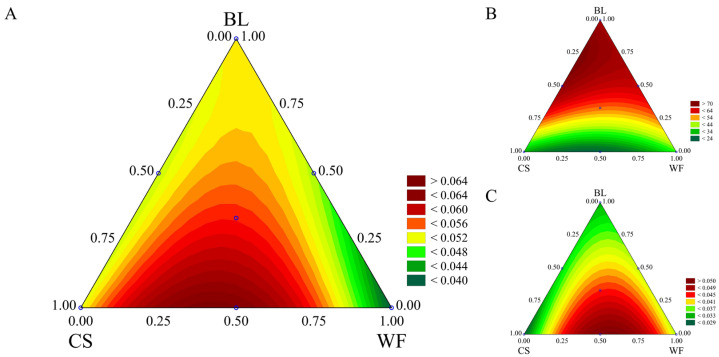
Contour plots obtained from the mixture design used to optimize the formulations with respect to thickness (**A**), water solubility (**B**), and water vapor permeability (**C**) of films prepared from corn starch (CS), wolf fruit (WF), and butterfly lily (BL), respectively. The blue point indicates the centroid (center point), corresponding to the mixture with equal proportions (1/3:1/3:1/3) of CS, WF, and BL.

The quadratic regression model showed a significant fit to the δ data (F = 138.02; *p* < 0.001), with an adjusted coefficient of determination (*R*^2^_*adj*._ = 0.972) (Table 3), explaining approximately 97.9% of the observed variability and demonstrating excellent predictive capability. All regression coefficients were statistically significant (*p* < 0.001), yielding Equation (1) and Figure 5A.

δ (mm) = 0.050 CS + 0.04 WF + 0.053 BL + 0.075 CS × WF − 0.007 CS × BL + 0.013 WF × BL
(1)

The highest δ value was observed for the CS:WF film (δ = 64.3 μm), suggesting a synergistic interaction between these two starches that results in a greater polymer matrix volume. The CS:WF:BL blend showed an intermediate thickness (δ = 56.5 μm), indicating that the balanced combination of the three starches produces films with moderate thickness.

#### 3.2.2. Water Solubility

The WS of the films ranged from 23.2 ± 0.4% to 72.8 ± 4.4% among the different treatments (Table 2). The BL film exhibited high WS (68.1 ± 5.2%), while the CS:BL blend showed the highest value (WS = 72.8 ± 4.4%), which may be related to the high AAC of BL (33.9%) and its lower short-range order (R_1047/1022_ = 0.931) compared to CS. Starches with a higher proportion of amorphous regions and less organized chains tend to exhibit greater solubility, as they facilitate water penetration and the leaching of starchy material during immersion [26,28]. Additionally, the presence of very long chains (DP > 37) in BL amylopectin, although contributing to the structural stability of the native granule, may not be sufficient to restrict solubilization when the starch is processed into films, especially in combination with CS.

In contrast, WF and CS:WF films showed the lowest WS values (39.2 ± 4.5% and 23.2 ± 0.4%, respectively). This behavior may be associated with the lower *δmax* of WF and its greater packing ability during film formation, resulting in a more compact matrix with reduced availability of chains for solubilization. Although WF presents the lowest short-range order (R_1047/1022_ = 0.929), which would theoretically favor solubility, the effect of granular packing appears to have been predominant, especially when combined with CS. This result suggests that, in binary systems, the interaction between granules of different sizes can generate denser arrangements that limit water penetration.

The CS:WF:BL blend showed intermediate WS (54.2 ± 2.0%), reflecting a balance among the characteristics of the three starches. This value is close to the weighted average of the individual solubilities (31.2% for CS, 39.2% for WF, and 68.1% for BL), suggesting that, in the ternary formulation, no significant synergistic or antagonistic interactions occurred to drastically alter the expected solubility.

It is noteworthy that the highest WS did not correspond to the film with the highest δ, indicating that WS is governed primarily by the chemical composition and molecular organization of the starches rather than solely by film thickness. The CS:BL blend combined the high AAC of BL with the ordered structure of CS, resulting in a possibly more heterogeneous matrix with greater release of soluble chains.

These results are consistent with findings reported in the literature, which demonstrate that starch solubility and that of their films are determined by the complex interaction among short-range order, amylopectin architecture, and granular morphology [25,26]. Additionally, they support the perspective that binary and ternary formulations can produce films with tunable solubility properties, enabling specific applications where greater or lesser interaction with water is desired.

The quadratic model adequately fitted the WS results (F = 53.04; *p* < 0.001; *R*^2^_*adj*._ = 0.967, Table 3), indicating excellent fit and high predictive capability (explaining approximately 97% of the variability), and showing that WS is influenced by both the individual proportions of the components and their interactions. All regression coefficients were statistically significant (*p* < 0.001), yielding Equation (2) of the quadratic model. The corresponding contour plot is shown in Figure 5B.

WS (%) = 31.35 CS + 39.35 WF + 68.25 BL − 50.98 CS × WF + 89.60 CS × BL + 44.42 WF × BL
(2)

The significant linear effect (*p* < 0.001) confirms that the AAC of each starch exerts a direct influence on the WS of the films, with BL (having the highest AAC = 33.9%) being primarily responsible for the increase in WS (Figure 5B), as observed in the BL (WS = 68.1%) and CS:BL (WS = 72.8%) treatments.

The significant quadratic effect, in turn, reveals that synergistic interactions among the starches also modulate this property, as evidenced by the low WS of the CS:WF film (WS = 23.2%), which cannot be explained solely by the linear combination of the components.

#### 3.2.3. Water Vapor Permeability

The WVP of the films ranged from 8.0 ± 0.4 10^−8^ to 12.9 ± 1.1 10^−8^ g (m s Pa)^−1^ among the different treatments (Table 2). The quadratic model adequately fitted these results, with an adjusted coefficient of determination (*R*^2^_*adj*._ = 0.540) (Table 3), explaining approximately 54% of the variability. In this case, WVP is influenced by the interactions among the components of the formulated blends. All regression coefficients were statistically significant (*p* < 0.001), yielding Equation (3) and its corresponding contour plot in Figure 5C, where the variability of WVP as a function of the starches used can be observed.

WVP [g (m s Pa)^−1^ 10^−8^] = 7.96 CS + 10.37 WF + 9.08 BL + 16.41 CS × WF (3)

The CS film exhibited the lowest WVP (8.0 ± 0.4 10^−8^ g (m s Pa)^−1^), which may be related to its high short-range order (R_1047/1022_ = 0.946) and A-type crystalline structure, typical of cereals. The literature indicates that matrices with higher molecular organization and dense packing of amylopectin chains tend to form more compact films, with a disordered pore distribution that hinders the diffusion of water molecules, resulting in lower WVP [25,26].

In contrast, the CS:WF blend film exhibited the highest WVP (12.9 ± 2.4 10^−8^ g (m s Pa)^−1^), associated with the combination of starches with different structural characteristics. The presence of WF, with its lower δ (7.3–15.8 μm) and low short-range order (R_1047/1022_ = 0.929), may have contributed to the formation of a matrix with discontinuities or regions of lower packing density, facilitating water vapor transport. Additionally, the CS:WF film showed the highest δ (64.3 ± 1.2 μm) among all treatments, which, although typically associated with increased vapor diffusion, may have been accompanied by a less cohesive structure that favored permeability.

The WF film presented intermediate WVP (10.5 ± 1.7 10^−8^ g (m s Pa)^−1^), higher than that of the CS film but lower than those of the CS:WF and CS:WF:BL blends. This result is consistent with the lower structural order of WF, which would theoretically favor higher WVP. However, its smaller *δmax* may have promoted more efficient packing during film formation, partially compensating for molecular disorder and resulting in a moderate water vapor barrier.

The BL film showed a WVP of 9.2 ± 1.1 10^−8^ g (m s Pa)^−1^, a value close to that of the CS film, despite its high AAC (33.9%) and lower short-range order (R_1047/1022_ = 0.931). This behavior may be partially explained by the presence of very long chains (DP > 37) in its amylopectin, typical of B-type crystalline starches, which act as structuring elements and confer greater cohesion to the film matrix—a trend observed in studies relating starch structural features (such as molecular distribution and FTIR band ratios) to physicochemical properties [27,29]. Additionally, the larger *δmax* of BL (12.6–55.5 μm) may have contributed to a matrix with less efficient packing but with long chains forming a more resistant network to water diffusion.

The ternary CS:WF:BL blends showed WVP of 11.9 ± 0.3 10^−8^ g (m s Pa)^−1^, a relatively high value close to that of the CS:WF blend (12.9 ± 2.4 10^−8^ g (m s Pa)^−1^). This suggests that the ternary combination may have generated a matrix with structural heterogeneities, in which different granular morphologies and molecular structures created interfaces that facilitate water vapor diffusion. The absence of a positive synergistic effect in reducing WVP indicates that the balance among components did not favor the formation of a more effective barrier.

This behavior reinforces that WVP is governed primarily by molecular organization, packing density, and interactions among starch chains, rather than solely by matrix thickness. Studies relating structural characteristics of different starches to their functionality show that molecular composition and chain arrangements strongly influence gelation and rheological properties [26,28].

These results are consistent with literature reports indicating that the WVP of starch-based films is strongly influenced by the molecular organization of the polymer matrix, amylose/amylopectin composition, and intermolecular interactions, rather than only by film thickness. The internal structure and packing density of the polymer network determine how easily water molecules diffuse through the film, reflecting the complex interplay among short-range order, chain chemistry, and morphological properties of the matrix [25,26]. Additionally, it has been shown that binary and ternary formulations of starches, plasticizers, and other components can tailor water barrier properties, enabling specific applications requiring higher or lower WVP depending on the intended use.

### 3.3. Physiological Quality of Common Bean Seeds

The regression model results for the QFS responses (Table 4) are presented in Table 5.

The fitted global regression models were highly significant for all observed responses, with calculated F values ranging from 16.27 to 106.32 (*p* < 0.001), indicating that the quadratic regression components satisfactorily described the behavior of the evaluated properties as a function of the blend composition.

The model fitted for MSP showed excellent predictive capability, with a non-significant lack of fit (F = 0.43; *p* > 0.05), confirming that the obtained regression equation adequately represents the variation of the experimental data within the studied domain. In contrast, the models fitted for GER, PC, and CP revealed a significant lack of fit (8.89 ≤ F ≤ 79.12; *p* = 0.001), indicating that the applied models may not be fully adequate to describe these properties. This suggests the need for further investigations, including the incorporation of higher-order (cubic) terms in the blend model or the inclusion of additional variables capable of capturing the complexity of the interactions involved in the formation of the polymer matrix and their influence on permeability.

#### 3.3.1. First Count

PC values ranged from 50.0% to 68.0% among the different starch blends used for seed coating. The highest seedling vigor was observed in seeds coated with pure BL (PC = 68.0%), followed by the CS:BL (PC = 67.5%) and WF:BL (PC = 63.5%) blends. The lowest PC performance was recorded for the CS:WF blend (50.0%), indicating that this combination of starches was unfavorable for germination speed.

**Figure 6:**
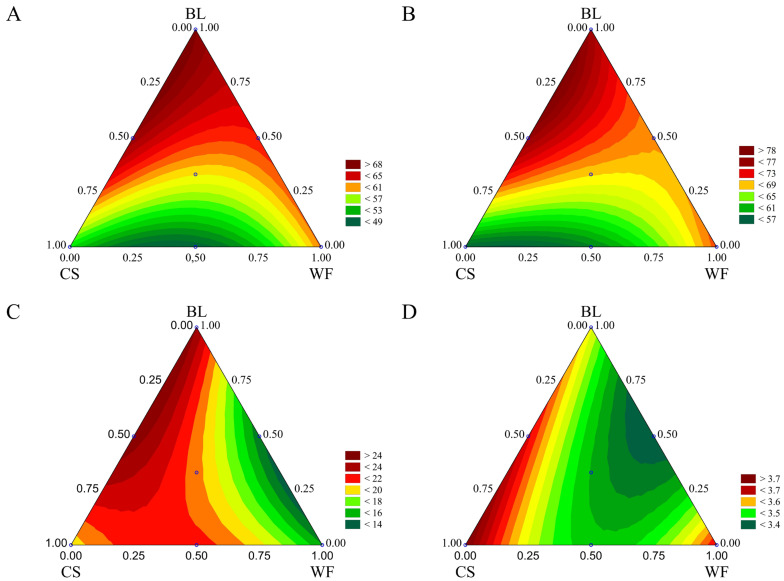
Contour plots obtained from the mixture design used to optimize formulations with respect to the quality of common bean seeds coated with corn starch (CS), wolf fruit (WF), and butterfly lily (BL): (**A**) first count, (**B**) germination, (**C**) seedling length, and (**D**) seedling dry mass. The blue point indicates the centroid (center point), corresponding to the mixture with equal proportions (1/3:1/3:1/3) of CS, WF, and BL.

Statistical analysis revealed that the quadratic model ($R_{adj.}^{2}$ = 0.946) was highly significant (F = 49.31; *p* < 0.01). However, the lack-of-fit test was significant, indicating that although the model explains 95.6% of the data variation, there are still unaccounted complexities not captured by the linear and quadratic terms, suggesting possible higher-order interactions among the components. The quadratic regression model and its corresponding contour plot are presented in Equation (4) and Figure 6A.

FC (%) = 54.19 CS + 61.18 WF + 68.18 BL − 33.56 CS × WF + 22.44 CS × BL − 7.56 WF × BL
(4)

The excellent performance of BL starch and the CS:BL blend can be directly related to the properties of the films formed by these materials. BL starch, as previously discussed, exhibits the highest gelatinization temperature (T_p_ = 77.52 °C), indicating greater thermal and structural stability of its granules. This characteristic results in films with a more organized and stable polymer matrix, as evidenced by its moderate water vapor permeability (WVP = 9.2 10^−8^ g (m s Pa)^−1^) and relatively high thickness (52.7 μm).

The CS:BL blend, in turn, produced films with the highest water solubility (72.8%) and intermediate permeability (8.4 10^−8^ g (m s Pa)^−1^), suggesting that this combination promotes a balance between the structural stability of BL and controlled hydration capacity. This balance likely favors rapid and uniform metabolic activation of seeds during imbibition, resulting in higher germination speed.

#### 3.3.2. Germinatiom

GER ranged from 55.5% to 76.5% among the treatments (Table 4). The highest germination percentage was observed in the BL treatment (76.5%), followed by the ternary blend CS:WF:BL (75.5%) and CS:BL (73.0%). The lowest value was recorded for CS:WF (55.5%), confirming the trend observed for vigor that this binary combination is the least suitable for seed coating.

The quadratic model ($R_{adj.}^{2}$ = 0.739) was significant (F = 8.89; *p* < 0.01) (Equation (5) and Figure 6B). Although it showed a better fit, the lack of fit indicates that the model may not fully capture the complexity of the interactions among blending components affecting the overall seed germination process.

GER (%) = 57.19 CS + 70.69 WF + 75.69 BL − 20.71 CS × WF + 39.29 CS × BL
(5)

The superior effect of BL on germination may be attributed to its ability to form films with adequate thickness and intermediate permeability, which likely create a microenvironment with balanced water and gas exchange, allowing complete hydration and embryonic development without restrictions. In contrast, the CS:WF:BL blend, which produced films with greater thickness and high permeability, showed higher germination (75.5%) due to differentiated water uptake conditions, favoring overall germination even with the intermediate vigor (57.5%) observed in the seed lots.

#### 3.3.3. Seedling Length

Seedling length ranged from 14.8 cm to 25.2 cm among the treatments (Table 4). The greatest growth was observed in seeds coated with the CS:BL blend (25.2 cm), followed by BL starch (23.9 cm) and the CS:WF blend (22.0 cm). The lowest seedling lengths were re-corded for WF alone (14.9 cm) and the WF:BL blend (14.8 cm), indicating that the pre-sence of WF starch, especially when combined with BL, restricts cell elongation and seedling growth.

Statistical analysis showed that the quadratic model ($R_{adj.}^{2}$ = 0.951) was highly significant (F = 79.12; *p* < 0.01) and provided an excellent fit, explaining 96% of the data variation (Equation (6) and Figure 6C). However, the lack-of-fit test was significant, indicating that despite the high coefficient of determination, the model may not be fully adequate for prediction, suggesting additional complexities in the interactions affecting growth.

SL (%) = 19.32 CS + 15.04 WF + 24.05 BL + 17.19 CS × WF + 11.97 CS × BL − 20.97 WF × BL
(6)

The good performance of the CS:BL blend for seedling length (CP = 25.2 cm) is consistent with its superiority in vigor, as also indicated by the first count test. This treatment combined the highest water solubility (72.8%) with the lowest permeability (8.4 10^−8^ g (m s Pa)^−1^), creating an optimal environment: high solubility allows rapid initial hydration (favoring emergence), while low permeability helps maintain stable conditions for continuous growth. The pure BL film, with its relatively high thickness (52.7 μm) and moderate permeability, also promoted seedling growth (23.9 cm). In contrast, films containing WF (either alone or combined with BL) produced the smallest seedlings, possibly due to the formation of denser or less permeable films (WF alone: WVP = 10.5 10^−8^; WS = 39.2%), which may have imposed mechanical resistance to radicle expansion or restricted the gas exchange required for metabolic growth processes.

#### 3.3.4. Seedling Dry Mass

SDM ranged from 3.4 g to 3.8 g (Table 4). The highest dry biomass accumulation was observed in seeds coated with CS (3.8 g), followed by the CS:BL blend (3.7 g) and pure WF (3.7 g). The lowest value was recorded for the WF:BL blend (3.4 g), indicating lower efficiency in assimilating partitioning or in early seedling development.

The quadratic model for seedling dry mass was highly significant (F = 28.68; *p* < 0.01), with a good fit ($R_{adj.}^{2}$ = 0.808) and a non-significant lack of fit (F = 0.43; *p* > 0.05) (Equation (7) and Figure 6D). This confirms that the quadratic model has predictive capability for this variable, adequately describing the behavior of dry mass as a function of blend composition.

SDM (%) = 3.76 CS + 3.65 WF + 3.56 BL − 0.98 CS × WF − 0.91 WF × BL
(7)

The higher dry mass accumulation promoted by CS (3.8 g) contrasts with its relatively lower vigor performance, according to the first count and germination results. This effect may be associated with the lower solubility of corn starch film (31.2%), which, although limiting initial germination, may have allowed a slower and more gradual water release, resulting in more robust seedlings at the end of development. Seedlings from the CS:BL blend also showed high dry mass (3.7 g), suggesting that, in addition to the excellent protection provided by this film, the combination of controlled permeability and high solubility favors adequate hydration without structural damage.

#### 3.3.5. Integrated Analysis of Germination and Seedling Development

**Figure 7:**
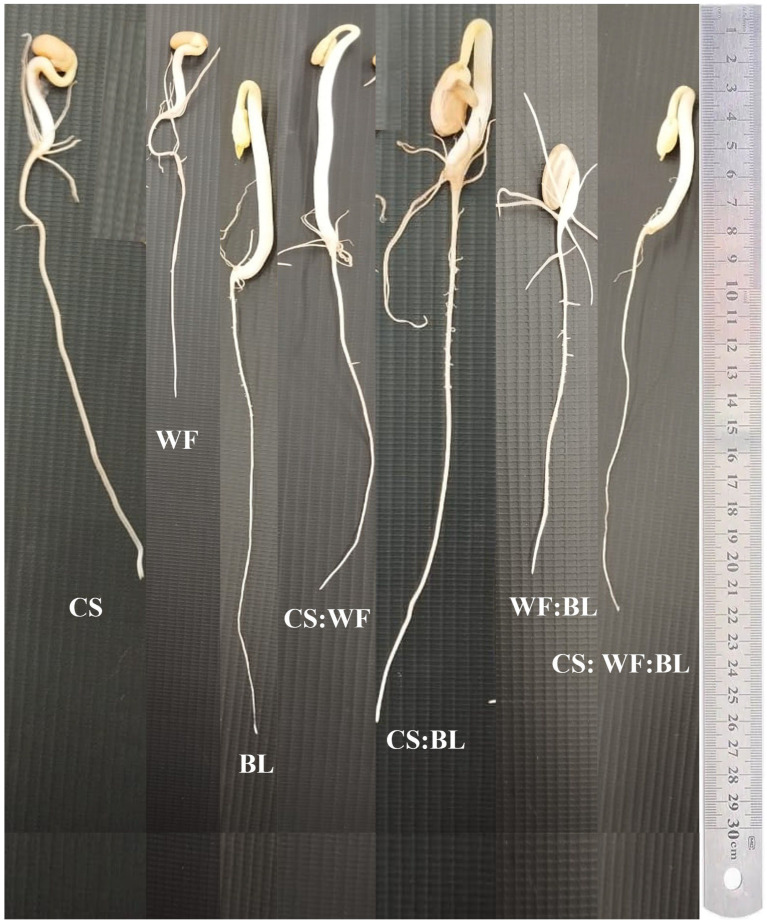
Normal common bean seedlings evaluated in the germination test, showing seedling length as a function of corn starch (CS), wolf fruit starch (WF), butterfly lily starch (BL), and their binary and ternary blends. Scale bar = X cm.

When all physiological parameters are considered together, a consistent pattern emerges. The BL treatment promoted the best overall performance, with the highest germination (76.5%), highest first count (68.0%), and second highest seedling length (23.9 cm), indicating high physiological vigor. The CS:BL blend showed remarkably superior seedling length (25.2 cm—the highest among all treatments), along with high germination (73.0%) and first count (67.5%), suggesting a synergistic effect between CS and BL on cell elongation and early seedling development. These differences are visually confirmed in Figure 7, which shows representative seedlings from each treatment.

In contrast, the CS:WF treatment exhibited the poorest germination (55.5%) and first count (50.0%), although it maintained a relatively high seedling length (22.0 cm). This suggests that while longitudinal development was not compromised, emergence capacity and biomass accumulation were negatively affected. The WF alone treatment resulted in intermediate germination (71.5%) but limited seedling length (14.9 cm), indica-ting that post-germinative growth was restricted. The WF:BL blend showed the lowest seedling length (14.8 cm) and dry mass (3.4 g), suggesting antagonistic interactions between these starches. The ternary treatment CS:WF:BL presented excellent germination (75.5%) but only intermediate seedling development, indicating that the addition of a third component did not provide additional benefits.

Taken together, the results demonstrate that BL is the most promising component for seed treatment, especially when combined with corn starch (CS:BL), enhancing root elongation and overall seedling vigor. The presence of WF in binary blends tends to limit post-germinative growth, except when combined with BL, where germination is maintained but development remains restricted. Simpler formulations (BL alone or CS:BL) were more effective than the ternary blend for seed treatment purposes.

## 4. Conclusions

According to the results, it was demonstrated that the characteristics of corn, wolf fruit, and butterfly lily starches directly influence the properties of the bioactive coatings and the physiological quality of common bean seeds. The characterization revealed that BL exhibits a higher apparent amylose content (33.9%) and larger granule diameter (26.11 µm), while WF shows a lower amylose content (22.7%) and higher peak viscosity (4646.74 cP), and CS presents a higher short-range molecular order (R_1047/1022_ = 0.946).

Films produced from the binary and ternary blends showed variable physical pro-perties, with emphasis on the CS:WF blend, which resulted in the greatest thickness (64.3 µm) and the lowest solubility (23.2%), and the CS:BL blend, which exhibited the highest solubility (72.8%). Water vapor permeability was mainly influenced by interactions among components, with the CS:WF blend being the most permeable (12.9 10^−8^ g (m s Pa)^−1^).

Regarding the physiological quality of common bean seeds, a positive synergistic effect was observed for the CS:BL blend, which provided high germination (73.0%), high vigor in the first count (67.5%), and the greatest seedling length (25.2 cm). BL starch alone also stood out, showing the highest germination (76.5%) and vigor (68.0%). In contrast, the CS:WF blend was detrimental, reducing germination (55.5%) and vigor (50.0%). Seedling dry mass was less influenced by the blends, ranging from 3.4 to 3.8 g.

In conclusion, the starches studied and their blends show potential as bioactive coa-tings. The CS:BL binary combination was the most promising for improving the physiological quality of common bean seeds, demonstrating a synergistic effect between corn starch and butterfly lily starch.

## Figures and Tables

**Table 1:** Physical and physicochemical properties of corn (CS), wolf fruit (WF), and butterfly lily (BL) starches.

Pararameters	CS	WF	BL
Size particle (*δmax*, µm)	10.54 ± 4.40	11.68 ± 2.38	26.11 ± 8.39
Amylose apparent content (%)	25.5 ± 0.6	22.7 ± 0.4	33.9 ± 1.2
R_1047/1022_	0.946	0.928	0.937
To (onset, °C)	61.47	58.12	71.52
Tp (Peak temperature, °C)	69.74	62.47	77.52
Tf (Endset, °C)	83.94	73.75	87.73
Tm (Menting peak, °C)	103.95	96.37	106.03
Enthalpy (J/g)	15.65–16.38	14.32–14.76	14.80–24.20
Pasting Temperature (°C)	67.98	67.71	79.44
Peak Viscosity (cP)	2698.54	4646.74	3087.06
Breakdown (cP)	1302.81	1791.74	798.2
Setback (cP)	2488.78	1144.40	6931.41

*δmax* = maximum diameter; R_1047/1022_ = crystallinity ratio in the wavenumber regions at 1047 and 1022 cm^−1^.

**Table 2:** Starch blend formulations obtained using a {3,2} simplex lattice design, and the mean values and standard deviations of film thickness (δ), water solubility (WS), and water vapor permeability (WVP) of the prepared films.

Blend	Starch			δ (μm)	WS (%)	WVP × 10^−8^ [g (m s Pa)^−1^]
	CS	WF	BL			
CS	1.0	0.0	0.0	51.3 ± 1.5	31.2 ± 3.0	8.0 ± 0.4
WF	0.0	1.0	0.0	39.7 ± 0.6	39.2 ± 4.5	10.5 ± 1.7
BL	0.0	0.0	1.0	52.7 ± 1.5	68.2 ± 3.7	9.2 ± 1.1
CS:WF	0.5	0.5	0.0	64.3 ± 1.2	23.2 ± 0.4	12.9 ± 2.4
CS:BL	0.5	0.0	0.5	50.3 ± 0.6	72.8 ± 4.4	8.4 ± 0.7
WF:BL	0.0	0.5	0.5	49.7 ± 2.1	65.5 ± 2.5	9.7 ± 1.6
CS:WF:BL	0.33	0.33	0.33	56.7 ± 1.5	54.2 ± 2.0	11.9 ± 0.3

CS, WF, BL = corn, wolf fruit, and butterfly lily, respectively.

**Table 3:** Summary of the analysis of variance (ANOVA) for the quadratic regression model applied to the results of thickness, water solubility, and water vapor permeability of the prepared films.

Parameters	Factor	MS	F	*R* ^2^_*adj*._
Thickness	Model	2.0 10^−4^	138.02 **	
	Lack of Fit	4.6 10^−7^	0.30 ^ns^	
	Quadratic	2.8 10^−4^	189.84 **	0.972
Water solubility	Model	1372.8	118.31 **	
	Lack of Fit	8.8	0.75 ^ns^	
	Quadratic	615.4	53.04 **	0.967
Water vapor permeability	Model	11.2	5.69 **	
	Lack of Fit	2.6	1.32 ^ns^	
	Quadratic	12.8	6.49 **	0.540

MS = mean square; F = F-value; $R_{adj.}^{2}$ = adjusted coefficient of determination; ns = not significant; ** *p* < 0.01.

**Table 4:** Results of the effect of starch coating on the physiological quality of common bean seeds, evaluated by germination (GER), first count (FC), seedling length (SL), and seedling dry mass (SDM) tests.

Blend	Starch			FC (%)	GER (%)	SL (cm)	SDM (g)
	CS	WF	BL				
CS	1.0	0.0	0.0	54.0 ± 1.4	58.0 ± 0.8	19.2 ± 1.8	3.8 ± 0.0
WF	0.0	1.0	0.0	61.0 ± 1.2	71.5 ± 1.3	14.9 ± 1.3	3.7 ± 0.1
BL	0.0	0.0	1.0	68.0 ± 0.8	76.5 ± 1.0	23.9 ± 2.2	3.6 ± 0.1
CS:WF	0.5	0.5	0.0	50.0 ± 1.6	55.5 ± 0.6	22.0 ± 5.3	3.5 ± 0.0
CS:BL	0.5	0.0	0.5	67.5 ± 1.3	73.0 ± 0.8	25.2 ± 1.4	3.7 ± 0.0
WF:BL	0.0	0.5	0.5	63.5 ± 1.3	66.0 ± 0.8	14.8 ± 1.7	3.4 ± 0.0
CS:WF:BL	0.33	0.33	0.33	57.5 ± 1.0	75.5 ± 0.6	19.3 ± 2.2	3.5 ± 0.1

CS, WF, BL = corn, Wolf fruit, and butterfly lily starches.

**Table 5:** Summary of the analysis of variance (ANOVA) for the linear and quadratic regression models applied to the results of first count, germination, seedling length, and seedling dry mass tests of common bean seeds.

Parameters	Factor	MS	F	$R_{adj.}^{2}$
FC	Model	216.995	95.99 **	
	Lack of Fit	16.734	10.65 **	
	Quadratic	2.261	49.31 **	0.946
GER	Model	270.762	16.27 **	
	Lack of Fit	350.189	459.62 **	
	Quadratic	16.645	8.89 **	0.739
SL	Model	78.462	106.32 **	
	Lack of Fit	8.292	21.92 **	
	Quadratic	0.738	79.12 **	0.951
SDM	Model	0.089	23.76 **	
	Lack of Fit	0.002	0.43 ^ns^	
	Quadratic	0.004	28.68 **	0.808

FC = first count; GER = germination; SL = seedling length; MSP = seedling dry mass; MS = mean square; F = F-value; $R_{adj.}^{2}$ = adjusted coefficient of determination; ns, not significant; ** *p* < 0.01.

## Data Availability

The raw data supporting the conclusions of this article will be made available by the authors on request.
